# Seaweed-Derived Extract Targets Porphyr’ageing to Modulate the Visible Signs of Aging in Human Skin

**DOI:** 10.3390/md24060220

**Published:** 2026-06-18

**Authors:** Morgane De Tollenaere, Marie Meunier, Emilie Chapuis, Marine Bracq, Cyrille Jarrin, Perrine Lemagnen, Patrick Robe, Laura Lapierre, Jean Tiguemounine, Catherine Zanchetta, Anne Humeau, Aurélie Préchoux, Jeremy Brebion, Franck Hennequart, Maud Benoit, Amandine Scandolera, Romain Reynaud

**Affiliations:** 1 Givaudan Active Beauty, Research and Development, Givaudan France SAS, 95018 Argenteuil, France; marie.meunier@givaudan.com (M.M.); emilie.chapuis@givaudan.com (E.C.); marine.bracq@givaudan.com (M.B.); cyrille.jarrin@givaudan.com (C.J.); perrine.lemagnen@givaudan.com (P.L.); patrick.robe@givaudan.com (P.R.); laura.lapierre@givaudan.com (L.L.); catherine.zanchetta@givaudan.com (C.Z.); anne.humeau@givaudan.com (A.H.); amandine.scandolera@givaudan.com (A.S.); romain.reynaud@givaudan.com (R.R.); 2 Plastic Surgery, Polyclinique Courlancy, 51100 Reims, France; jean.tigue@me.com; 3 ALGAIA R&D Center, 50000 Saint-Lô, France; aurelie.prechoux@algaia.com (A.P.); jeremy.brebion@algaia.com (J.B.); franck.hennequart@algaia.com (F.H.); maud.benoit@algaia.com (M.B.)

**Keywords:** laminaran, laminarin, coproporphyrin, collagen, elastin, wrinkle, pigmentation, melanin

## Abstract

Recent evidence suggests that microbiota-derived porphyrins contribute to skin aging, a phenomenon termed porphyr’aging. These pro-inflammatory molecules alter the expression of genes involved in senescence, trigger melanogenesis, and decrease collagen I synthesis in skin. The aim of this study was to evaluate the anti-aging properties of an upcycled *Laminaria hyperborea* extract (LHE) targeting bacterial porphyrins discovered after screening. The impact of LHE on porphyrin biosynthesis and on melanogenesis and wrinkles was evaluated using *in vitro* and *ex vivo* tests and by conducting a double-blinded *vs*. placebo clinical trial. LHE significantly reduced coproporphyrin III production in Gram-positive skin bacteria and significantly decreased porphyrin levels *in vivo* at the skin surface. This activity was supported by a specific composition of LHE, comprising laminaran and mannitol. It also significantly decreased melanin content in skin explants and pigmentation in the clinical study (−5.9%). This effect was particularly pronounced in dark spots (ITA +39.9%), and the number of precursor spots also decreased (−6.9%). In addition, LHE significantly stimulated type I α-1 pro-collagen production in fibroblasts and increased collagen I and elastin expression in skin explants. These results were consistent with the clinical study, showing significant reductions in wrinkle number (−9.8%) and area (−5.8%). These findings suggest that targeting microbiota-derived porphyrins and their consequences may represent a promising approach to reduce the visible signs of aging.

## 1. Introduction

Ingredients of natural origin are in demand in the cosmetic industry to address environmental concerns and consumer health. Marine resources are a reservoir of unique metabolites for the development of innovative and effective ingredients, of which algae are the most promising organisms for cosmetic applications [1]. Seaweeds and micro-algae are widely distributed photosynthetic organisms with a renewable character. Due to their richness in polysaccharides, polyphenols, proteins, lipids, or carotenoids [1,2], they offer multifunctional benefits, such as skin soothing or lightening properties [3,4,5].

*Phaeophyceae* (brown seaweed) are multicellular algae, among which Laminariales, also known as kelp, form large organisms living in coastal rocky habitats in temperate and Arctic regions [6]. Several species of kelp are of economic importance for food, especially in the hydrocolloid industry (alginates) and aquaculture, and have found applications in cosmetics [6]. This is the case of *Undaria pinnatifida* and the *Laminaria* sp. *L. digitata*, *L. japonica* and *L. ochroleuca* [5,7]. For example, fucoxanthin from *L. japonica* reduces skin pigmentation in animal models, and its phlorotannins act as UV protectors [7,8]. Kelp polysaccharides, fucoidans and laminarins, also called laminarans, display a wide range of bioactivities, of which antioxidant and anti-inflammatory effects could be of interest for cosmetic applications [9,10].

Aging is a complex process resulting from the interplay of several internal and external factors, such as genetics, diet, sunlight, and exposure to pollution [11]. At the cellular level, hallmarks of aging characterize the events causing senescence [12], leading to the expression of an aged skin phenotype: hyperpigmentation or hypopigmentation, wrinkles, skin thinning, or loss of elasticity. In addition to the major drivers of skin aging, mainly UV exposure, our team recently demonstrated that porphyrins, metabolites produced by the skin microbiota, could also play a role in this process [13]. Porphyrins are ubiquitous molecules, typical of skin-inhabiting microbes. The major porphyrin detected at the healthy skin surface is coproporphyrin III [14,15,16], which is commonly associated with the presence of *Cutibacterium acnes* [17,18]. However, other genera within the cutaneous microbiota, such as *Corynebacterium* and *Staphylococcus*, are also capable of synthesizing this compound [19,20]. These fluorescent molecules can be easily observed on the human face, especially in pilosebaceous units [14,17]. Our team recently evidenced that microbial porphyrins could contribute to skin aging [13]. In this preliminary work, we showed that porphyrins penetrate the *stratum corneum* and reach the living epidermis. In cellular models, such compounds elicited melanin synthesis, reactive oxygen species, pro-inflammatory interleukin-8, and decreased collagen production, at the root of aging. Porphyrins caused the down-regulation of several genes involved in cell proliferation, DNA repair and integrity, or cell senescence, possibly interfering with skin homeostasis and reinforcing the hypothesis that microbial molecules have a role in the appearance of the aged phenotype. *In vivo*, they were positively and significantly correlated with hyperpigmentation and wrinkles—the major visible characteristics of aging—so this phenomenon was qualified as porphyr’ageing. These data suggest that targeting bacterial porphyrins could decrease hyperpigmentation and wrinkles in human skin.

*L. hyperborea* extract (LHE) was selected from several natural extracts for its ability to inhibit porphyrin production by skin bacteria. In this study, we investigated the potential of *Laminaria hyperborea*, an underexplored kelp species in the cosmetic industry, as an anti-aging active that modulates microbiota-derived porphyrins—also referred to as “Porphyr’ageing”. LHE was evaluated for the reduction of porphyrin synthesis in skin bacteria and *in vivo*, and its effects on the clinical signs of aging—dark spots and wrinkles—were assessed.

## 2. Results

### 2.1. Characterization of LHE Specific Composition

**Figure 1:**
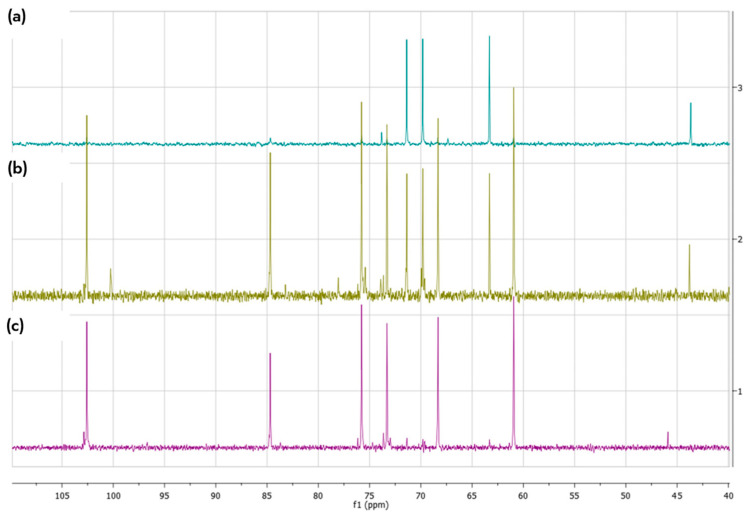
^13^C NMR spectra in deuterium oxide of (**a**) the LHE ultrafiltration permeate containing low molecular weight molecules, (**b**) LHE, and (**c**) a commercial reference of laminaran from *Laminaria digitata*.

LHE is derived from laminaran-rich (>45%) seaweed *L. hyperborea;* complete characterization remains confidential. A ^13^C NMR structural analysis was conducted on LHE and a commercial laminaran from *L. digitata*, prepared in deuterium oxide (D_2_O). Recorded spectra are presented in Figure 1. Comparison revealed a correspondence between the 6 major chemical shifts of LHE (60.93, 68.32, 73.30, 75.78, 84.68, 102.57 ppm) with *L. digitata* laminaran, corresponding to the carbon signals from the glucose units in the polysaccharidic chains. Signals with chemical shifts at 63.30, 69.80, and 71.36 ppm were characteristic of D-mannitol (CAS 69-65-8) in D_2_O (Figure 1a) and are not found in *L*. *digitata* (Figure 1c), and suggest that this main component of LHE differs from the composition of *L. digitata*. LHE was then evaluated for its anti-aging properties in human skin.

### 2.2. LHE Modulates Porphyrin Content In Vitro and In Vivo

**Figure 2:**
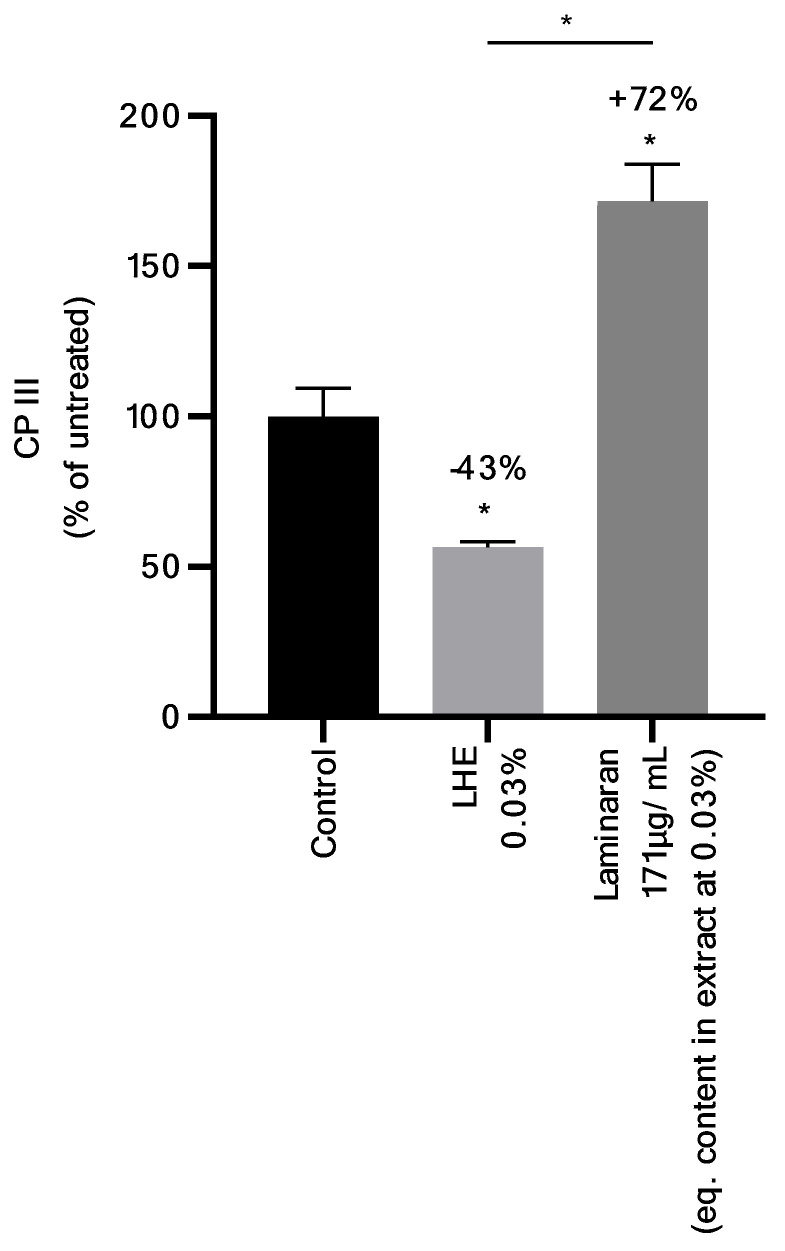
Quantification of porphyrins in the culture medium of three skin bacterial strains after incubation with LHE 0.03% or laminaran from *Laminaria digitata* at 171 µg/mL, equivalent to the polysaccharide content in LHE. Results are expressed as a percentage of untreated, mean ± SEM. Statistical analysis was performed using the Mann–Whitney test. * *p* < 0.05.

Three *Cutibacterium acnes* strains isolated from human skin were selected to represent the genetic diversity of this species. The strains belong to different phylogenetic clusters, as demonstrated by their clade, ribotype, and SLST type (Table 1). They were cultivated in RCM medium with and without LHE 0.03%, and CPIII was quantified in the culture medium (Figure 2). In the presence of LHE, the content of extracellular CPIII decreased, but not with the commercial laminaran from *Laminaria digitata* tested at the equivalent dose (171 µg/mL) in the LHE at 0.03%, suggesting that the unique composition of LHE modulated CPIII metabolism.

**Figure 3:**
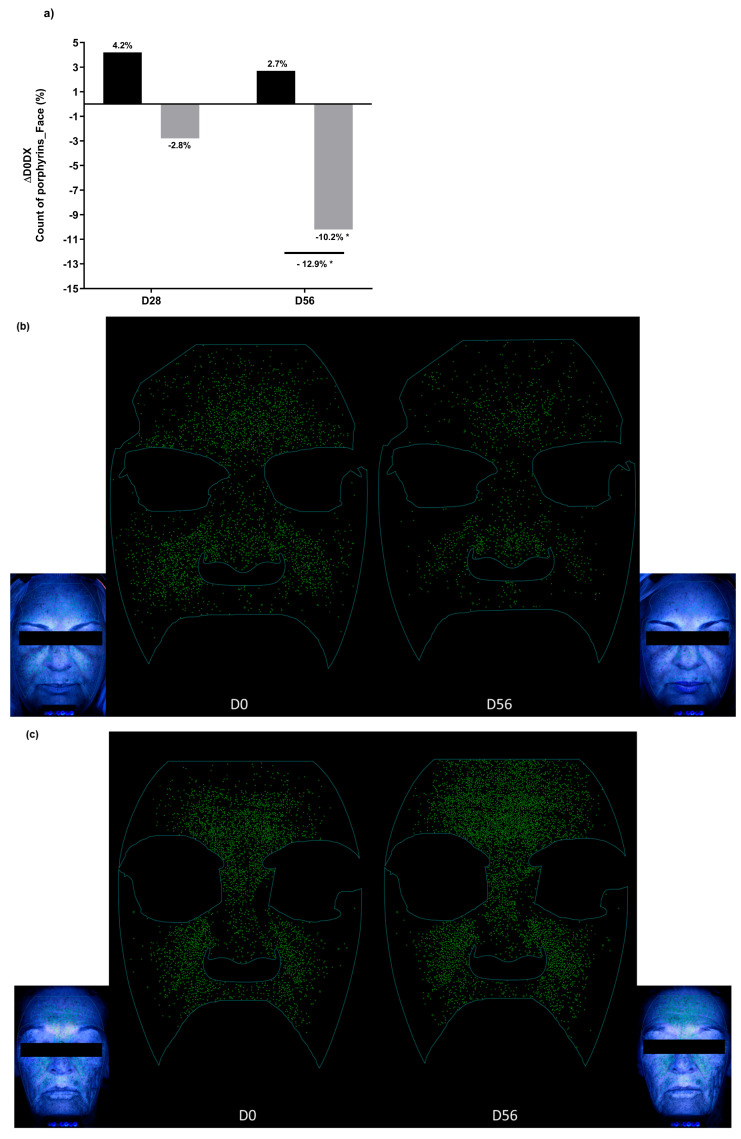
Determination of facial porphyrins in women volunteers with VISIA CR2.3^®^. (**a**) Quantification of porphyrin counts in the whole face of participants after 28 and 56 days, vs. day 0, following twice daily application of a placebo cream (black bars) or containing 0.03% of LHE (grey bars). (**b**,**c**) Illustrative pictures of the porphyrin fluorescence in the whole face of two volunteers at day 0 (D0) and after 56 days (D56) of twice daily application of 0.03% LHE (**b**) or with placebo cream (**c**). Statistical analysis conducted with paired *t*-test vs. D0 and with unpaired *t* test vs. placebo. * *p* < 0.05.

Then, the effect of LHE on porphyrin content was directly quantified *in vivo*. Using VISIA^®^-CR 2.3 (Canfield Scientific, Parsippany, NJ, USA), porphyrin counts were measured across the entire face of volunteers who applied a placebo cream or an identical formula containing LHE 0.03% for 56 days (Figure 3). Porphyrin count decreased by −2.8% and significantly by −10.2% after 28 and 56 days, respectively, compared to D0, and reached a significant variation of −12.9% vs. placebo after 56 days.

**Figure 4:**
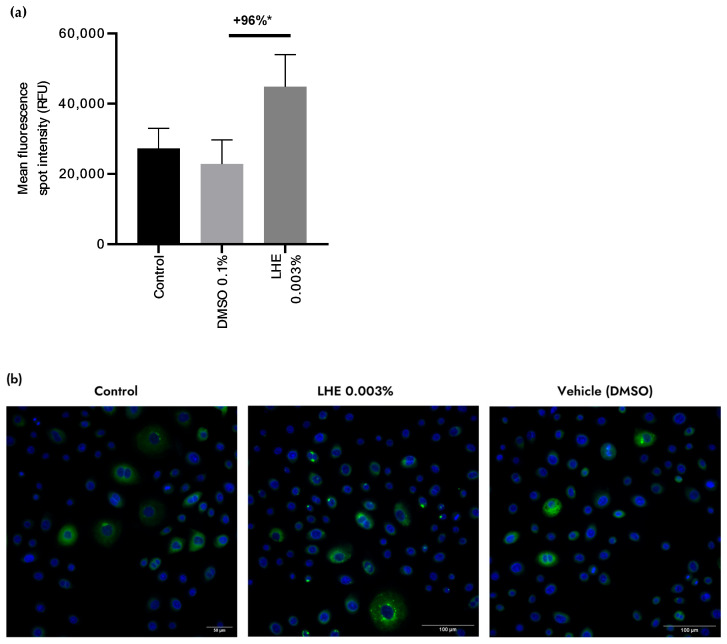
Estimation of lysosomal activity in normal human epidermal keratinocytes (NHEKs). (**a**) Quantification of lysosomal activity in NHEKs after 30 h of treatment with LHE 0.003%. Results are mean of fluorescence intensity ± SEM. (**b**) Illustrative pictures of fluorescent labeling of lysosomal activity (blue: nuclei, green: lysosomes). Statistical analysis was performed using the Mann–Whitney test. * *p* < 0.05.

CPIII has the capacity to penetrate human skin [13], and we were interested in lysosomal activity as a possible degradative pathway for these metabolites in keratinocytes. Lysosomes are acidic organelles containing hydrolases that require low pH for their optimal activity. They ensure the degradative function of macromolecules and damaged proteins and organelles, a process called autophagy that decreases with aging [21,22]. The effect of LHE 0.003% on the lysosomal activity of keratinocytes was quantified (Figure 4). LHE increased the lysosomal activity significantly compared to the untreated control and the vehicle control (+96%). This may imply more efficient recycling activities within the cells.

### 2.3. LHE Reduces Pigmentation Ex Vivo and In Vivo

**Figure 5:**
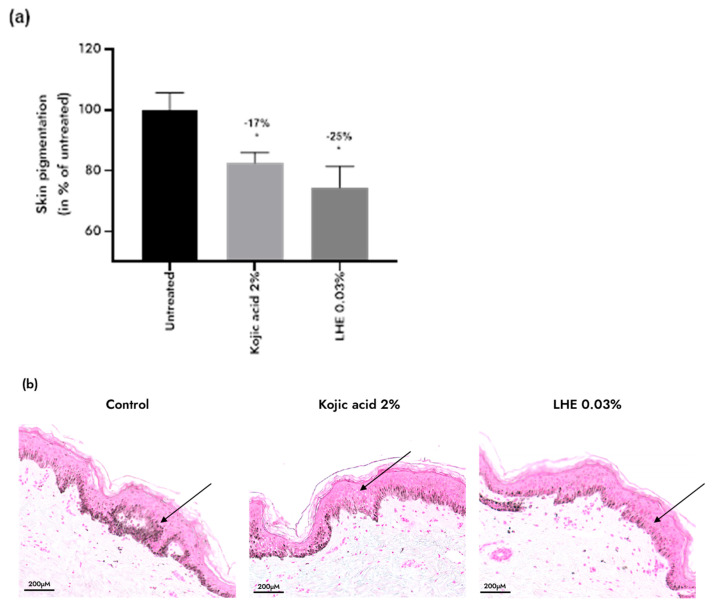
Assessment of melanin content in the epidermis of skin explants of a 36-year-old donor by Fontana–Masson staining. (**a**) Quantification of melanin content in skin explants untreated, or treated with kojic acid 2% (positive control), or with LHE 0.03%. Results are the mean staining intensity of melanin, normalized to the untreated control, in % ± SEM. The percentage of reduction is calculated by subtraction from the control (**b**). Illustrative pictures of Fontana–Masson staining of skin explants from the three conditions previously described. Black arrow indicates melanin. Statistical analysis was performed using the Mann–Whitney test. * *p* < 0.05.

Pigmentation disorders—uneven skin tone, age, and dark spots—constitute visible signs of ageing. The ability of LHE to reduce melanin in skin explants was measured using Fontana–Masson staining (Figure 5). Kojic acid was used as a positive control. It significantly reduced melanin content by −17%, and the LHE condition reached −25% of pigmentation. These results indicate that LHE can directly act on melanogenesis or melanin degradation.

**Figure 6:**
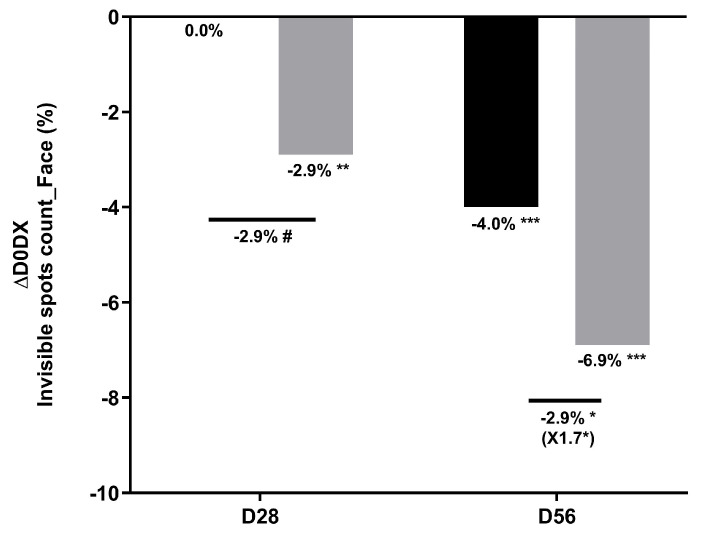
Quantification of invisible spots in the whole face of women volunteers after 28- and 56-days vs. day 0, following twice daily application of a placebo cream (dark bars) or a cream containing 0.03% of LHE (grey bars). Statistical analysis was conducted using a paired *t*-test vs. D0 and an unpaired *t*-test vs. placebo: ^#^ *p* < 0.1, * *p* < 0.05, ** *p* < 0.01, *** *p* < 0.001.

The clinical evaluation of the LHE effect on facial skin pigmentation analyzed the invisible spots with VISIA-CR 2.3^®^ (Figure 6). Invisible spots correspond to the accumulation of melanin within the epidermis that is not yet visible to the human eye, but that can be detected under ultraviolet light. They can be considered as the precursors of dark spots [13]. The number of invisible spots was significantly reduced from day 28 in the group applying LHE 0.03% cream, with significant results vs. placebo (−2.9%) after 56 days.

**Figure 7:**
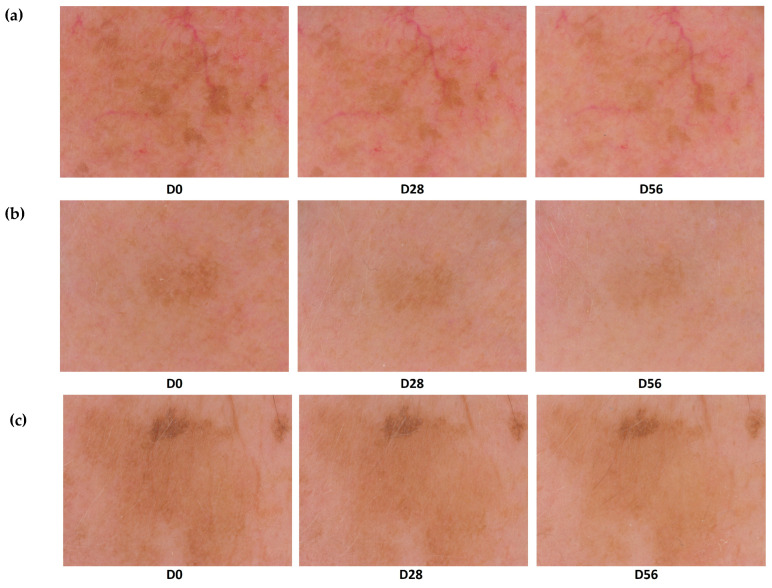
Illustrative picture of three women volunteers of a selected spot area at day 0 (D0) and after 28 (D28) or 56 (D56) days of twice daily application of (**a**,**b**) a cream containing LHE 0.03% or (**c**) a placebo cream, on the whole face.

Visible skin pigmentation was assessed in participants by measuring the individual typology angle (ITA) on a selected spot area and the overall melanin content (Table 1). After 56 days of application, the group applying the LHE-containing formula presented a higher ITA in the hyperpigmented zones and lower melanin content. A higher ITA value indicates a decrease in dark spot pigmentation, as illustrated by the picture presented in Figure 7, and that is consistent with the reduction of melanin content.

### 2.4. LHE Promotes Extracellular Matrix Protein Expression and Improves Skin Relief and Texture

**Figure 8:**
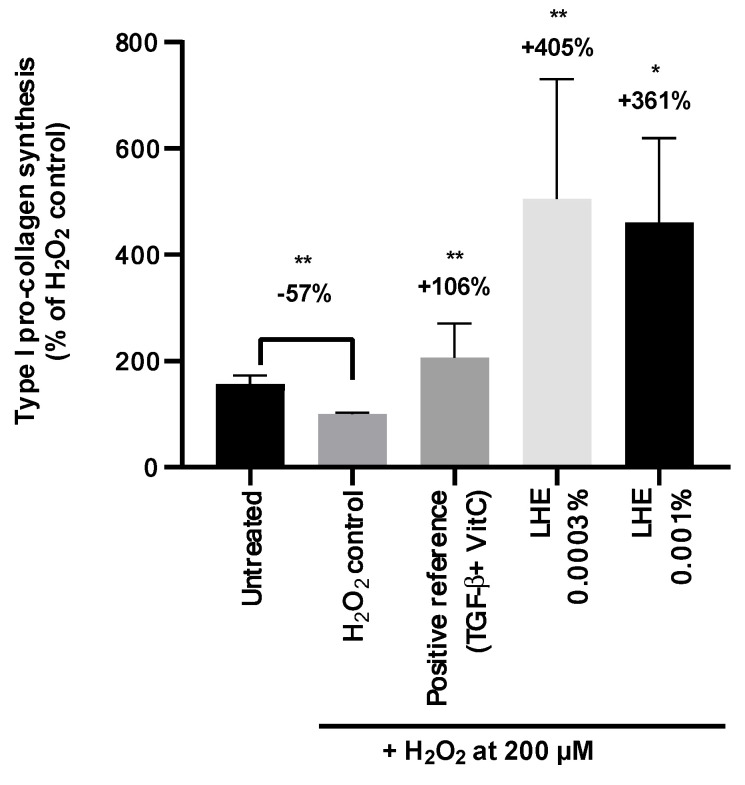
Quantification of type I α-1 pro-collagen in normal human dermal fibroblasts (NHDFs) untreated or under H_2_O_2_-induced ageing alone or in combination with LHE 0.003% or 0.001%. TGFβ and vitamin C were used as positive controls for the induction of type I α-1 pro-collagen protein expression. Results are expressed in % relative to the H_2_O_2_ control mean ± SEM. Statistical analysis was performed using the Mann–Whitney test: * *p* < 0.05, ** *p* < 0.01.

**Figure 9:**
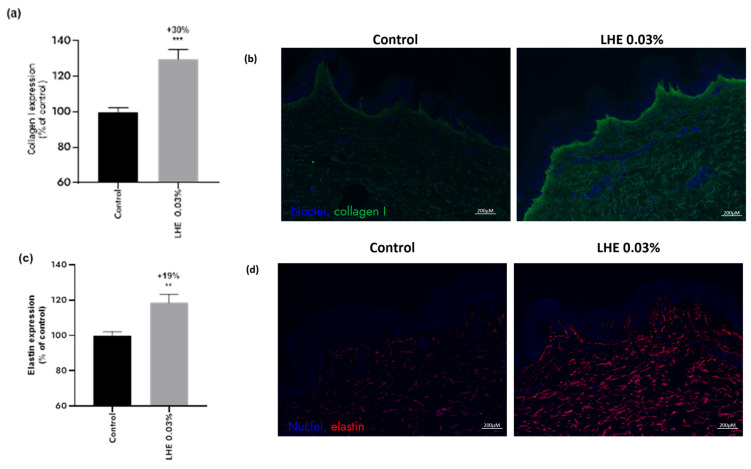
Analysis of two components of the extracellular matrix in human skin explants of a 56-year-old donor. (**a**) Collagen I protein expression determination in skin explants, untreated or treated with LHE 0.03%, and determined from the fluorescence intensity of collagen I labeling. (**b**) Illustrative pictures of skin explant slices with fluorescent detection of nuclei (blue) and collagen I (green). (**c**) Elastin protein expression determination in skin explants, untreated or treated with LHE 0.03%, and determined from fluorescence intensity of elastin labeling. (**d**) Illustrative pictures of skin explant slices with fluorescent detection of nuclei (blue) and elastin (red). Results are mean ± SEM. Statistical analysis was performed using the Mann–Whitney test. ** *p* < 0.01 and *** *p* < 0.001.

Another important visible aspect of skin aging is the appearance of wrinkles, associated with a degradation of the extracellular matrix components in the dermis. The impact of LHE on two important components of the extracellular matrix, collagen I and elastin, was first evaluated on fibroblasts and skin explants. In a model of aged fibroblasts mimicked by the hydrogen peroxide (H_2_O_2_) stress, the stress control showed a significant reduction of pro-collagen I α-1 expression in comparison to the untreated control (−57%). The LHE-treated condition, stressed with hydrogen peroxide, showed a significant increase in pro-collagen I α-1 synthesis (Figure 8). In skin explants from a 56-year-old female donor, both collagen I and elastin protein expression increased significantly by +30% (Figure 9a) and +19% (Figure 9c), respectively.

**Figure 10:**
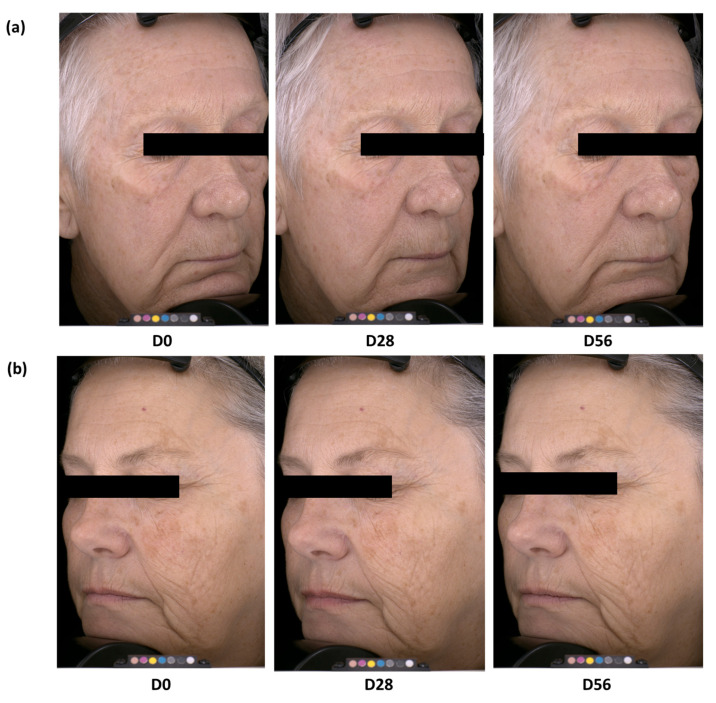
Illustrative picture of the whole face of two women volunteers at time 0 and after 28 or 56 days of twice daily application of (**a**) a cream containing LHE 0.03% or (**b**) a placebo cream.

The clinical investigation revealed that the group applying LHE cream had fewer wrinkles than the placebo group, with a significant reduction in wrinkle count by −5.5% after 56 days (Table 2). Wrinkle area also decreased by −5.8% after 56 days with a significant value vs. placebo. Both fine and coarse lines presented a lower area from day 28. These results are illustrated by pictures presented in Figure 10. LHE improved skin texture, with a progressive decline of roughness on day 28 by −3.1% (placebo −0.6%), and on day 56 by −6.4% (placebo −3.5%).

## 3. Discussion

The use of marine ingredients in anti-aging cosmetic formulas is on the rise, particularly for actives extracted from algae [23]. Algae constitute the main source of marine poly-saccharides [1], of which *Phaeophyceae* (brown algae) contain alginate, marine cellulose, fucoidan and laminaran [10]. In this work, we explored the potential of LHE, derived from *L. hyperborea*, as an anti-aging active for human skincare targeting porphyr’aging.

LHE was selected among other natural extracts as a potential modulator of porphyrin synthesis. In the present work, LHE significantly decreased CPIII production in the culture medium of three Gram-positive bacteria strains isolated from skin. Compared with a commercial laminaran from *L. digitata*, LHE specifically modified the CPIII metabolism of the bacteria. The characterization of LHE by ^13^C NMR revealed the presence of a polysaccharide close to laminaran and of mannitol. Laminarans, also called laminarins, are low-molecular-weight β-glucan, composed of β-1,3-linked D-glucose units, with some β-1,6 branches [24]. The structure of laminarans varies depending on seaweed species, season, or environmental factors, and influences their physicochemical properties and bioactivities [24]. The specific activity of LHE could be attributed to its unique composition, including mannitol. Christensen et al. showed that laminaran from *L. hyperborea* differs from that of *L. digitata* in terms of β-1,6 linkage percentage, at 4% and 10%, respectively. This difference allows *L. hyperborea* laminaran only to influence cytokine secretion in dendritic cells [25]. The anti-inflammatory effect of LHE was confirmed in preliminary screening studies, which showed that LHE significantly reduced the release of IL-6 and TNF-α. (Supplementary Figures S1 and S2). Since porphyrins demonstrated local pro-inflammatory stress, it would be interesting to conduct further studies in these specific porphyrins-mediated-inflammatory conditions [13].

The double-blinded vs. placebo clinical study was conducted on 37 women aged 45–73 years. Skin physiology in terms of ageing being different between men and women, we selected only women to ensure standardization and homogeneity [26]. Nonetheless, it would be interesting to conduct a similar study in a male cohort. In this work, the *in vivo* study revealed LHE reduced skin porphyrins content *in vivo*. This ability thus may rely on the negative regulation of LHE on the bacterial metabolism of CPIII. Moreover, a higher lysosomal activity was observed in keratinocytes treated with LHE. As we previously demonstrated, porphyrins deposited at the skin’s surface can penetrate up to the living epidermis [13]. Although the degradative pathway of porphyrins is not yet described in skin cells, several works observed them to localize in lysosomes [27,28]. Structural characteristics of anionic porphyrins, such as coproporphyrin, make them more prone to localize in the acidic compartment of the lysosome [29]. Autophagy appears as a possible degradative pathway but further work should investigate more thoroughly the exact pathway of degradation. Nonetheless, it cannot be excluded that a higher lysosomal activity could have promoted a more efficient clearance of bacterial porphyrins.

Because porphyrins are significantly and positively correlated with hyperpigmented spots and wrinkles *in vivo* [13], the ability of LHE to mitigate these important signs of skin aging is also interesting. In addition to this correlation, bacterial porphyrins are known to increase oxidative stress and promote melanin synthesis in keratinocyte-melanocyte co-culture [13]. In the present work, LHE reduced melanin content in phototype V skin explants; this effect was confirmed *in vivo* across various skin phototypes (II-III). Pigmentation decreased specifically in the dark spot area, and LHE also reduced the invisible precursors of dark spots. To our knowledge, *L. hyperborea* has not previously been reported to have a hypopigmenting effect on human skin. Brown algae metabolites, such as phlorotannins, meroterpenoids, fucoxanthin, or fucoidans, have demonstrated potential anti-melanogenic properties [4,8]. A deeper analysis of these molecules in LHE should be conducted in future studies to further investigate their possible role in the regulation of pigmentation. Nevertheless, Laminaran from *L. digitata* and *L. hyperborea*, as well as mannitol, are described as possessing antioxidant properties that reduce oxidative stress in skin cells [30,31,32]. In a preliminary study, we demonstrated that LHE exhibited antioxidant activity in skin cells (Supplementary Figure S3), consistent with the literature. This antioxidant activity can be clearly linked to the melanogenesis inhibition effect of LHE observed in this work. Indeed, it is well described that melanin synthesis is an oxidation reaction, and oxidative stress has an impact on melanogenesis-related proteins, stimulating pigment synthesis [33].

The weakening of the dermis is at the root of wrinkle formation. In aging, the extracellular matrix (ECM) network, composed of collagen and elastic fibers embedded in glycosaminoglycan-rich proteoglycan, undergoes dramatic changes. The amount of collagen, the major component of ECM, decreases, reducing interactions between the ECM and fibroblasts and leading to impaired cellular function [34]. Bacterial porphyrins inhibit the type I α-1 collagen expression in fibroblasts [13]. Elastic fibers are degraded as well, and the renewal of the degenerated matrix decreases [34]. In this work, LHE was demonstrated to increase the expression of type I α-1 pro-collagen in prematurely aged fibroblasts and of collagen I and elastin proteins in skin explants. By promoting the synthesis of the elementary components of the dermal matrix, LHE could improve its renewal. It is an independent benefit that can be added to the counteracting effect against the negative influence of bacterial porphyrins over type I α-1 collagen synthesis in fibroblasts [13]. These *in vitro* and *ex vivo* data support the results of the *in vivo* study: in the group using the cream composed of LHE, the number and the area of wrinkles were reduced on both coarse and fine lines. Brown seaweeds have already been described to have anti-aging properties. For example, sulfated polysaccharides rich in fucose are known to down-regulate matrix metalloproteinase involved in collagen degradation [9]. Nevertheless, the laminaran from *L. digitata* was reported to not affect type I α-1 pro-collagen content in normal human fibroblasts [30], suggesting that LHE—that increased the protein expression in H_2_O_2_-aged fibroblasts—has unique collagen-promoting activity.

## 4. Conclusions

This work underscores the potential of *L. hyperborea* for designing anti-aging cosmetics using a novel approach. We developed a strategy targeting the porphyr’aging by using LHE to decrease microbiota-derived porphyrins in skin. From cellular models to clinical trials, LHE mitigated two important visible signs of aging correlated with porphyrin content: hyperpigmentation and wrinkles. As an upcycled ingredient from seaweed, LHE is an example of a more sustainable ingredient developed from marine sources, answering the actual demand for natural-origin and science-backed actives.

## 5. Materials and Methods

### 5.1. Seaweed-Derived Extract Preparation and Characterization

*Laminaria hyperborea* extract (LHE) is derived from the laminaran-rich brown seaweed of the Laminariaceae family. The extract is a co-product of agricultural crop-ingredient manufacturing. Fresh algae sustainably collected in France are extracted and turned into powder using a solvent-free process. Briefly, fresh brown seaweed biomass (*Laminaria hyperborea*) is cut and extracted in acidic aqueous media at 70 °C using a 1% citric acid solution for 3 h under stirring conditions. Then, a centrifugation step is performed to remove solid residues. The supernatant is recovered and filtered a second time, followed by a concentration step to remove water. The recovered precipitate is dewatered a second time to obtain a 10%DM paste. Finally, this paste is spray-dried to obtain laminaran extract in powder form. The extract is rich in laminaran (>45%), which was characterized by ^13^C NMR (Nuclear Magnetic Resonance). Briefly, samples were dissolved in 99.9% D_2_O (Thermo Fisher Scientific, Waltham, MA, USA) at a concentration of 8 mg/mL. The products were then transferred into a 5-mm NMR tube, and ^13^C NMR spectra were recorded at 70 °C using a 500 MHz BRUKER Avance III HD spectrometer (Bruker, Billerica, MA, USA). The solvent resonance was used as the internal standard. Data processing was performed by MestReNova software, version 14.3.3 (Mestrelab Research, Santiago de Compostela, Spain). Chemical shifts were expressed in parts per million (ppm).

### 5.2. Quantification of Porphyrins in Cutibacterium acnes Culture Medium

*C. acnes* strains were purchased from BEI Resources (Manassas, VA, USA) and are described in Table 3. The strains are reference genomes for the Human Microbiome Project and were isolated from human skin.

Bacteria were seeded from a working cell bank (50 µL) into 20 mL of Reinforced Clostridium Medium (RCM) and cultured anaerobically for 3 days with stirring (130 rpm) at 37 °C in a single sealed bag containing Anaerocult^®^ P (Merck, Darmstadt, Germany) and an Anaerotest^®^ strip (Merck, Darmstadt, Germany). A volume of the preculture was transferred to a 50 mL Erlenmeyer flask containing 20 mL of RCM in order to obtain a starting OD600 nm of 0.020. The culture was continued in an anaerobic jar with GasPak™ EZ (Thermo Fisher Scientific) and an Anaerotest^®^ strip (Merck, Darmstadt, Germany). The culture medium was supplemented with 215 µL of LHE 30 mg/mL in DMSO, with a final concentration of 0.03%, or 215 µL of laminaran from *Laminiria digitata* (Merck, Darmstadt, Germany) in DMSO at 17 mg/mL with a final concentration of 170 µg/mL. A control culture (with DMSO only) was also prepared. Bacterial growth was assessed by measuring the optical density of culture samples at 600 nm.

After 24 h or 48 h, an aliquot of bacterial culture (1.3 mL) was collected and centrifuged at 18,000× *g*, 10 °C for 15 min. Supernatant (1 mL) was collected, and Coproporphyrin III (CPIII) was then extracted by liquid/liquid extraction with ethyl acetate/acetic acid (4:1, *v*/*v*) following a procedure adapted from Hamblin et al. [37]. The upper phase (2.4 mL) was collected, transferred to a new tube, and evaporated to dryness at 30 °C under vacuum (Genevac™; Fisher Scientific, Portsmouth, NH, USA). Dried extracts were suspended in 70 µL HCl 1.5 M.

CPIII quantification was adapted from Mancini et al. [38]. Samples were analyzed on an LC-MS UltiMate™ 3000 system (Thermo Fisher Scientific, Waltham, MA, USA) comprising a quaternary pump (LPG 3400 SD), an autosampler (WPS3000), and UV and MS detectors (DAD 3000 and ISQ EC, respectively). The system was controlled by Chromeleon 7.210ES software (Thermo Fisher Scientific, Waltham, MA, USA). Chromatographic separation on a Luna PFP column 5 µm, 100 Å, 4.6 × 150 mm (Phenomenex; Sigma-Aldrich, St. Louis, MO, USA) was achieved with a 2-solvent gradient. Solvent A was 0.1% formic acid in ultrapure water; solvent B was 0.1% formic acid in acetonitrile. The gradient applied was as follows: 0 min, 25% B; 2 min, 25% B; 15 min, 66% B; 15.5 min, 95% B; 19.5 min, 95% B; 20–24 min, 25% B. Flow was maintained at 1 mL/min, column oven temperature was 25 °C, and sampler temperature was set to 10 °C. CPIII elution was detected at 405 nm. The ISQ EC mass spectrometer was operated in full scan negative mode at 100–1000 Da with the following parameters: CID 20 V, dwell scan time 2 s, SIM widths 0.1 amu, sheet gas pressure 80 psi, auxiliary gas pressure 9.7 psi, sweep gas pressure 0.5 psi, and vaporizer temperature 550 °C. CPIII content was calculated by comparing peak intensity to a calibration curve of CPIII (MedChem Express, Monmouth Junction, NJ, USA) from 0.061 µM to 61.1 µM in HCl 1.5 M.

### 5.3. Evaluation of Lysosomal Activity in Keratinocytes

The test was carried out on normal human epidermal keratinocytes (NHEKs) isolated from biopsies. LHE was dissolved in DMSO at 10% with sonication for 15 min at 37 °C. Afterward, LHE was diluted in culture medium and tested at 0.003%, which is the highest non-cytotoxic dose (75% of viability relative to control after 48 h of incubation). The dose was determined using an MTT (3-[4,5-dimethylthiazol-2-yl]-2,5 diphenyl tetrazolium bromide) assay and morphological analysis [39]. NHEKs were seeded on 96-well plates (100 μL/well–6000 cells/well). After 24 h of culture, cells were treated with LHE at 0.003% and incubated for 30 h at 37 °C with 5% CO_2_. After 30 h of treatment, the medium was removed, and cells were incubated for 1 h with the Lysotracker L7528 probe (Thermo Fisher Scientific), which labels acidic compartments, mainly composed of lysosomes. Lysosomes were observed and quantified by automated fluorescence microscopy using the Cell Insight CX7 High-Content Screening Platform (Thermo Fisher Scientific).

### 5.4. Melanin Quantification in Skin Explants

Skin explants were obtained with the informed consent of a 36-year-old female donor undergoing abdominal surgery, with phototype V skin. Skin phototype is obtained by measuring the Individual Typology Angle (ITA) degree (according to Chardon et al., 1991 [40]) using SkinColorCatch (Delfin Technologies Ltd., Kuopio, Finland). A phototype II is needed as an inclusion criterion at least to observe a whitening effect at the *ex vivo* scale. The explants were kept alive by culturing on biocompatible plastic grids in standard 24-well plates at an air–liquid interface with skin culture medium (Givaudan, Vernier, Switzerland) at 37 °C and 5% CO_2_. The culture medium and treatment were renewed every 24 h. Skin explants were topically treated with LHE at 0.03% or kojic acid (Merck) at 2%. The active was pre-diluted at 1% in DMSO, then in the culture medium. Skin explants treated with sterile water were used as a negative control. After 5 days of treatment, the skin explants were fixed in 10% formalin for Fontana–Masson staining. The skin explants fixed in formalin 10% were dehydrated and embedded in paraffin. Slices of 4 µm thickness were cut and then dewaxed to be stained by the Fontana–Masson silver method for melanin visualization. Two images per explant were collected with an automated bright-field and digital confocal imaging system (ImageXpress^®^ PICO, Molecular Devices, version 2.10). Photomicrographs of Fontana–Masson-stained tissue sections were treated with GIMP (GNU Image Manipulation Program), a community-free software (https://gimp.org, (accessed on 13 September 2024) to extract the pixel area of melanin. Generated images were then analyzed using ImageJ open-source software [41] (v1.52a, https://imagej.net/ij/ (accessed on 13 September 2024) for quantification. The image was inverted, and the mean intensity was measured.

### 5.5. Quantification of Type I Pro-Collagen in Fibroblasts

Normal Human Dermal Fibroblasts (NHDFs) were seeded in 6 well-plates at 100,000 cells per well in triplicate. The cells were incubated for 48 h in complete medium (DMEM medium, Gibco, London, UK) supplemented with 10% fetal calf serum (FCS, Biowest, Nuaillé, France) and 1% antibiotics (Merck) at 37 °C with 5% CO_2_. After 48 h, cells in the “prematurely-aged” condition were stressed with hydrogen peroxide (H_2_O_2_, Sigma-Aldrich) at 200 µM for 2 h at 37 °C in 5% CO_2_. Cells were then rinsed twice with PBS (Gibco) and incubated for 48 h in basal medium (DMEM medium without FCS) supplemented with 1% antibiotics. Untreated cells received no treatment. The H_2_O_2_-stressed control cells received no further treatments. The other conditions were treated with LHE at 0.0003% and 0.001%, non-cytotoxic doses determined by the MTT assay (0.01% was the highest non-cytotoxic dose, with 89% viability relative to untreated), or with the positive reference: TGF-β and ascorbic acid (Merck). LHE was first diluted at 10 mg/mL in DMSO, then in basal medium. At the end of the culture, the media were collected and centrifuged at 2000× *g* for 10 min at 4 °C. The media were stored at −20 °C until pro-collagen I dosage. A crystal violet assay was performed to determine cell density. After collecting culture medium, cells were fixed with methanol (Merck) and incubated for 20 min at RT. Fixed cells were then rinsed two times with distilled water baths and allowed to dry. Adherent cells were then stained with crystal violet solution (Merck) for 15 min at RT. Then, the crystal violet solution was removed, and the stained cells were rinsed twice with distilled water baths and allowed to dry. A volume of 1 mL of acetic acid at 10% (Merck) was added into each well to dissolve stained cells. Homogenization was done under orbital agitation for a few minutes. The optical density was measured at a wavelength of 560 nm.

Type I α-1 pro-collagen quantification was performed with the Human Pro-Collagen I alpha 1 ELISA Kit (Abcam, Cambridge, UK). Briefly, supernatants were diluted at 1:200 in sample diluent except for those from the untreated condition, which were diluted at 1:300. The samples and the standard range were incubated for 1 h in 96-well plates pre-coated with type I pro-collagen antibodies in the presence of the cocktail antibody. After three washes with the wash buffer provided by the supplier, a TMB (Tetramethylbenzidine) substrate solution was added to the wells for 10 min in the dark. The catalysis of this substrate by horseradish peroxidase generates a blue color in proportion to the amount of type I pro-collagen bound in the initial step. The color development was stopped by a stop solution and changed to yellow. The optical density was measured at 450 nm and 540 nm with a microplate reader (TECAN SPARK^®^ 10 M, TECAN, Männedorf, Switzerland). Finally, the Four Parameter Logistic Curve analysis was performed via the MyAssays website (http://myassays.com, accessed on 19 May 2021) after subtraction of optical density at 450 nm from optical density at 540 nm. The quantification was normalized with the optical density measured by the crystal violet assay.

### 5.6. Quantification of Type Collagen-I and Elastin in Skin Explants

Skin explants were obtained with informed consent from a 56-year-old female donor undergoing abdominal surgery. The explants were kept alive by culturing on biocompatible plastic grids in standard 24-well plates in an air–liquid interface with skin culture medium (Givaudan) at 37 °C and 5% CO_2_. The culture medium was renewed every 24 h. Skin explants were topically treated with LHE at 0.03% in emulsion versus a placebo. After 4 days, the skin explant surfaces were rinsed with sodium phosphate buffer (PBS), then fixed in formalin, then dehydrated and embedded in paraffin for immunostaining.

Slices of 4 µm thickness were cut and dewaxed, and antigenic retrieval was performed overnight at 62 °C in citrate buffer pH 8. Non-specific sites were saturated with BSA 2% in Tris buffer, and the primary antibody was then incubated on skin slices overnight at 4 °C (anti-collagen I antibody 1:50, Abcam or anti-elastin antibody 1:75, Santa Cruz, Dallas, TX, USA). Next day, the excess of antibody was washed three times with Tris buffer and secondary antibody was incubated for 1 h at RT: for collagen I it was the Hoechst 33342 1:5000 coupled to Alexa fluor 488 anti-rabbit 1:200 and for elastin it was Hoechst 33342 1:5000 coupled to Alexa fluor 568 anti-rabbit 1:200. Excess antibodies were washed three times with Tris buffer and mounting medium without DAPI was added with coverslips. Pictures of the emitted fluorescent signal were taken with an inverted epifluorescence microscope (Axio Observer, Zeiss, Oberkochen, Germany). Fluorescence intensity for each condition was measured using ImageJ open-source software [41], and results obtained with LHE were compared with the untreated condition, considered the 100% control.

### 5.7. Clinical Evaluation of the Impact of LHE on Porphyrins and Aging Signs

The clinical study was double-blind and placebo-controlled. It was conducted on a panel of 37 women (skin phototypes II-III) aged between 45 and 73 years (average 65.3 ± 4.5 years old) presenting wrinkles, spot precursors, and pigmented spots on the face. The main inclusion criteria were:

- Women aged 45 to 75 years old;
- Women volunteers with wrinkles on the face and the neckline;
- Women volunteers with brown spots on the face and the neckline;
- Women with phototypes I, II, or III.

The panel was divided into two groups: group 1 consisted of 18 volunteers who applied a cream containing LHE at 0.03%, and group 2 consisted of 19 volunteers who applied a placebo cream (same formula without LHE). The INCI formula was: AQUA/WATER, CETYL ALCOHOL, GLYCERYL STEARATE, PEG-75 STEARATE, CETETH-20, STEARETH-20, ISODECYL NEOPENTANOATE, ± LAMINARIA HYPERBOREA EXTRACT, PHENOXYETHANOL, DIMETHICONE, FRAGRANCE. Application was performed twice daily (morning and evening) for 56 days to the whole face.

Porphyrin area and count, wrinkles, and precursor spots were quantified using VISIA^®^-CR 2.3 (Canfield Scientific, Parsippany, NJ, USA) by taking digital photographs of the face from the front and side at different times, with repositioning at D0. According to the VISIA^®^ manufacturer, porphyrins were visualized under red-lighting modality, and invisible spots under blue-light excitation.

The study protocol was approved by the Institutional Review Board of Givaudan (IRB No. 2022-004, approved in June 2022). Informed consent was obtained from all subjects involved in the study.

No adverse effects were observed during the study.

### 5.8. Statistical Analysis

*In vivo* studies were analyzed using a Shapiro–Wilk test to verify whether the raw data followed the Gaussian distribution. In case of normally-distributed data, the mean values were compared using either an unpaired or paired Student *t* test. In case of non-normally-distributed data, a Wilcoxon test was used for paired data, and a Mann–Whitney U test was used for unpaired data. Regardless of the test, results were considered significant at: *p* < 0.1 with ^#^, *p* < 0.05 with *, *p* < 0.01 with **, and *p* < 0.001 with ***.

## 6. Patents

Patent pending.

## Figures and Tables

**Table 1:** Analysis of facial skin pigmentation of women volunteers. Determination of ITA (individual typology angle) on spot area by C-Cube^®^ (C-Cube Microsystems, Milpitas, CA, USA), and melanin content by SiAscope^®^ (MedX Health Corp., Mississauga, ON, Canada), in the facial skin of participants at day 0 and after 28 or 56 days of twice daily application of a placebo cream (dark bars) or a cream containing 0.03% of LHE. Statistical analysis by paired *t*-test vs. D0 and unpaired *t*-test vs. placebo with: ^#^ *p* < 0.1, * *p* < 0.05, *** *p* < 0.001, ns: not significant.

	Placebo			LHE 0.03%			Unpaired *t* Test LHE 0.03% Versus Placebo (*p*)
	Mean +/− SEM (Arbitrary Unit)	Average Variation (%) vs. D0	Paired *t* Test Versus D0 (*p*)	Mean +/− SEM (Arbitrary Unit)	Average Variation (%) vs. D0	Paired *t* Test Versus D0 (*p*)	
ITA (C-Cube^®^)							
D0	18.2 ± 4.2			17.8 ± 5.4			
D28	21.3 ± 4.1	17.0%	***	21.6 ± 4.4	29.4%	***	ns
D56	23.5 ± 4.5	21.4%	***	24.9 ± 3.7	39.9%	***	*
Melanin content (SiAscope^®^)							
D0	238.9 ± 11.8			241.7 ± 24.4			
D28	237.5 ± 13.3	−0.6%	ns	237.6 ± 21.6	−1.7%	#	ns
D56	231.8 ± 11.4	−3.0%	***	227.4 ± 21.1	−5.9%	***	*

**Table 2:** Analysis of wrinkles and roughness in the facial skin of women volunteers. Determination of total wrinkle count and area, followed by fine and coarse lines area, and roughness, on day 0 and after 28 or 56 days of twice daily application of a placebo cream or a cream containing LHE 0.03%. Statistical analysis by paired t-test and Wilcoxon test with: ^#^ *p* < 0.1, * *p* < 0.05, ** *p* < 0.01, *** *p* < 0.001, ns: not significant.

	Placebo			LHE			Unpaired *t* Test LHE 0.03% Versus Placebo (*p*)
	Mean +/− SEM (Arbitrary Unit)	Average Variation (%) vs. D0	Paired *t* Test Versus T0 (*p*)	Mean +/− SEM (Arbitrary Unit)	Average Variation (%) vs. D0	Paired *t* Test Versus D0 (*p*)	
Wrinkle count							
D0	349.3 ± 69.1			365.7 ± 99.5			
D28	347.4 ± 75.9	−0.6%	ns	351.1 ± 92.8	−4.0%	*	ns
D56	334.4 ± 69.2	−4.3%	*	329.72 ± 89.4	−9.8%	***	*
	**Placebo**			**LHE**			**Mann Whitney test LHE 0.03%** **versus** **placebo (*p*)**
	**Mean +/− SEM** **(arbitrary unit)**	**Average** **variation (%) vs. D0**	**Wilcoxon test versus D0 (*p*)**	**Mean +/− SEM** **(arbitrary unit)**	**Average** **variation (%) vs. D0**	**Wilcoxon test versus D0 (*p*)**	
Wrinkle area							
D0	569.9 ± 106.3			584.8 ± 171.2			
D28	563.3 ± 109.6	−1.2%	ns	571.0 ± 153.2	−2.4%	#	ns
D56	569.8 ± 107.1	0.0%	ns	551.0 ± 157.1	−5.8%	**	*
Fine lines area							
D0	138.5 ± 45.6			151.2 ± 68.1			
D28	147.1 ± 52.7	6.2%	ns	142.2 ± 67.5	−6.0%	*	**
D56	133.3 ± 46.2	−3.8%	ns	135.7 ± 64.2	−10.3%	**	#
Coarse lines area							
D0	663.4 ± 118.8			718.7 ± 234.9			
D28	657.6 ± 129.7	−0.9%	ns	681.6 ± 228.4	−5.2%	**	**
D56	635.8 ± 101.1	−4.2%	ns	661.5 ± 218.3	−8.0%	***	#
Roughness							
D0	6.35 ± 0.63			6.63 ± 0.33			
D28	6.31 ± 0.48	−0.6%	*	6.43 ± 0.34	−3.1%	***	**
D56	6.13 ± 0.46	−3.5%	***	6.21 ± 0.33	−6.4%	***	**

**Table 3:** Characteristics of *C. acnes* strains involved in the assay. SLST: single-locus Sequence Typing.

Strain	Genome Coding	Clade [35]	Clade [36]	SLST Type [35]	Ribotype [35]
HM504	HL030PA1	IB	IB3	H1	RT1
HM523	HL038PA2	IA1	IB1	E1	RT8
HM525	HL059PA1	IA2	IB2	F1	RT16

## Data Availability

The original contributions presented in this study are included in the article; further inquiries can be directed to the corresponding author.

## Supplementary Materials

The following supporting information can be downloaded at: https://www.mdpi.com/article/10.3390/md24060220/s1, Figure S1. Quantification of IL-6 release in Normal Human Epidermal Keratinocytes (NHEKs) after 24 hours of pro-inflammatory stress mediated by Phorbol 12-myristate 13-acetat at 10ng/mL or in combination with LHE 0.0003%. Results are expressed in % relative to the stressed control mean ± SEM. Statistical analysis was performed using Mann Whitney test. * *p* < 0.05; Figure S2. Quantification of TNF-α release in Normal Human Epidermal Keratinocytes (NHEKs) after 24 hours of pro-inflammatory stress mediated by Phorbol 12-myristate 13-acetat at 10ng/mL or in combination with LHE 0.0003%. Results are expressed in % relative to the stressed control mean ± SEM. Statistical analysis was performed using Mann Whitney test. * *p* < 0.05; Figure S3. Quantification of intracellular Reactive Oxygen Species (ROS) in normal human skin cells untreated or after oxidative stress mediated by tert-Butyl hydroperoxide solution (TBP) at 5mM or in combination with LHE 0.0003% or 0.0001%. Resveratrol at 200µM was used as positive control. The ROS production was evaluated by measuring the fluorescence generated via the presence of the 2′,7′-Dichlorofluorescin diacetate (DCFH-DA) probe. Results are expressed in % relative to the stressed control mean ± SEM. Statistical analysis was performed using Mann Whitney test. * *p* < 0.05.

## Abbreviations

The following abbreviations are used in this manuscript:

CPIII	Coproporphyrin III
ITA	Individual Typology Angle
LHE	*Laminaria hyperborea* Extract
NHEK	Normal Human Epidermal Keratinocyte
NHDF	Normal Human Dermal Fibroblasts

## References

[1] Fonseca S., Amaral M.N., Reis C.P., Custódio L., Fonseca S., Amaral M.N., Reis C.P., Custódio L. (2023). Marine Natural Products as Innovative Cosmetic Ingredients. Mar. Drugs.

[2] López-Hortas L., Flórez-Fernández N., Torres M.D., Ferreira-Anta T., Casas M.P., Balboa E.M., Falqué E., Domínguez H., López-Hortas L., Flórez-Fernández N. (2021). Applying Seaweed Compounds in Cosmetics, Cosmeceuticals and Nutricosmetics. Mar. Drugs.

[3] Scandolera A., Hubert J., Humeau A., Lambert C., De Bizemont A., Winkel C., Kaouas A., Renault J.-H., Nuzillard J.-M., Reynaud R. (2018). GABA and GABA-Alanine from the Red Microalgae *Rhodosorus marinus* Exhibit a Significant Neuro-Soothing Activity through Inhibition of Neuro-Inflammation Mediators and Positive Regulation of TRPV1-Related Skin Sensitization. Mar. Drugs.

[4] Meunier M., Bracq M., Chapuis E., Lapierre L., Humeau A., Bernard S., Lambert C., Paulus C., Auriol P., Lemagnen P. (2023). Targeting SDF-1 as an Efficient Strategy to Resolve Skin Hyperpigmentation Issues with *Himanthalia elongata* Extract. J. Cosmet. Dermatol..

[5] Hernández Muñoz A.C., Rodríguez Martínez I.A., Serafini M.R., Aragón D.M. (2024). Innovative Applications of Marine-Derived Algae in Cosmetics: A Patent Review (2010−2023). Algal Res..

[6] Bolton J.J. (2010). The Biogeography of Kelps (Laminariales, Phaeophyceae): A Global Analysis with New Insights from Recent Advances in Molecular Phylogenetics. Helgol. Mar. Res..

[7] Kalasariya H.S., Pereira L., Kalasariya H.S., Pereira L. (2022). Dermo-Cosmetic Benefits of Marine Macroalgae-Derived Phenolic Compounds. Appl. Sci..

[8] Azam M.S., Choi J., Lee M.-S., Kim H.-R., Azam M.S., Choi J., Lee M.-S., Kim H.-R. (2017). Hypopigmenting Effects of Brown Algae-Derived Phytochemicals: A Review on Molecular Mechanisms. Mar. Drugs.

[9] Pangestuti R., Shin K.-H., Kim S.-K., Pangestuti R., Shin K.-H., Kim S.-K. (2021). Anti-Photoaging and Potential Skin Health Benefits of Seaweeds. Mar. Drugs.

[10] Li Y., Zheng Y., Zhang Y., Yang Y., Wang P., Imre B., Wong A.C.Y., Hsieh Y.S.Y., Wang D., Li Y. (2021). Brown Algae Carbohydrates: Structures, Pharmaceutical Properties, and Research Challenges. Mar. Drugs.

[11] Wong Q.Y.A., Chew F.T. (2021). Defining Skin Aging and Its Risk Factors: A Systematic Review and Meta-Analysis. Sci. Rep..

[12] Jin S., Li K., Zong X., Eun S., Morimoto N., Guo S. (2023). Hallmarks of Skin Aging: Update. Aging Dis..

[13] Meunier M., De Tollenaere M., Jarrin C., Chapuis E., Bracq M., Lapierre L., Zanchetta C., Tiguemounine J., Scandolera A., Reynaud R. (2025). Bacterial Porphyrins in Healthy Skin: Microbiota Components Impact Melanogenesis and Age-Related Processes Leading to Porphyr’ageing. Int. J. Cosmet. Sci..

[14] Patwardhan S.V., Richter C., Vogt A., Blume-Peytavi U., Canfield D., Kottner J. (2017). Measuring Acne Using Coproporphyrin III, Protoporphyrin IX, and Lesion-Specific Inflammation: An Exploratory Study. Arch. Dermatol. Res..

[15] Schaller M., Loewenstein M., Borelli C., Jacob K., Vogeser M., Burgdorf W.H.C., Plewig G. (2005). Induction of a Chemoattractive Proinflammatory Cytokine Response after Stimulation of Keratinocytes with *Propionibacterium acnes* and Coproporphyrin III. Br. J. Dermatol..

[16] Goerz G., Link-Mannhardt A., Bolsen K., Zumdick M., Fritsch C., Schürer N.Y. (1995). Porphyrin Concentrations in Various Human Tissues. Exp. Dermatol..

[17] McGinley K.J., Webster G.F., Leyden J.J. (1980). Facial Follicular Porphyrin Fluorescence: Correlation with Age and Density of *Propionibacterium acnes*. Br. J. Dermatol..

[18] Koenig K., Schneckenburger H., Hemmer J., Tromberg B.J., Steiner R.W. (1994). In-Vivo Fluorescence Detection and Imaging of Porphyrin-Producing Bacteria in the Human Skin and in the Oral Cavity for Diagnosis of Acne Vulgaris, Caries, and Squamous Cell Carcinoma. Proceedings of the Advances in Laser and Light Spectroscopy to Diagnose Cancer and Other Diseases.

[19] Alves G.B., da Costa Marques Calderari M.R., da Fonseca E.N., dos Santos L.S., de Mattos-Guaraldi A.L. (2024). Porphyrin Production by *Corynebacterium diphtheriae* Strains from Clinical Isolates. Chem. Biodivers..

[20] Wu J., Chu Z., Ruan Z., Wang X., Dai T., Hu X. (2018). Changes of Intracellular Porphyrin, Reactive Oxygen Species, and Fatty Acids Profiles During Inactivation of Methicillin-Resistant *Staphylococcus aureus* by Antimicrobial Blue Light. Front. Physiol..

[21] Tan J.X., Finkel T. (2023). Lysosomes in Senescence and Aging. EMBO Rep..

[22] He Y., Fan Y., Ahmadpoor X., Wang Y., Li Z.A., Zhu W., Lin H. (2024). Targeting Lysosomal Quality Control as a Therapeutic Strategy against Aging and Diseases. Med. Res. Rev..

[23] Resende D.I.S.P., Ferreira M., Magalhães C., Sousa Lobo J.M., Sousa E., Almeida I.F. (2021). Trends in the Use of Marine Ingredients in Anti-Aging Cosmetics. Algal Res..

[24] Liu Z., Xiong Y., Yi L., Dai R., Wang Y., Sun M., Shao X., Zhang Z., Yuan S. (2018). Endo-β-1,3-Glucanase Digestion Combined with the HPAEC-PAD-MS/MS Analysis Reveals the Structural Differences between Two Laminarins with Different Bioactivities. Carbohydr. Polym..

[25] Christensen M.D., Allahgholi L., Dobruchowska J.M., Moenaert A., Guðmundsson H., Friðjónsson Ó., Karlsson E.N., Hreggviðsson G.Ó., Freysdottir J. (2025). Laminarins and Their Derivatives Affect Dendritic Cell Activation and Their Crosstalk with T Cells. Int. J. Biol. Macromol..

[26] Luebberding S., Krueger N., Kerscher M. (2013). Skin Physiology in Men and Women: In Vivo Evaluation of 300 People Including TEWL, SC, Hydration, Sebum Content and Skin Surface PH. Intern. J. Cosmet. Sci..

[27] Strømhaug P.E., Berg T.O., Berg K., Seglen P.O. (1997). A Novel Method for the Study of Autophagy: Destruction of Hepatocytic Lysosomes, but Not Autophagosomes, by the Photosensitizing Porphyrin Tetra(4-Sulphonatophenyl)Porphine. Biochem. J..

[28] Gaullier J.-M., Gèze M., Santus R., Melo T.S.E., Mazière J.-C., Bazin M., Morlière P., Dubertret L. (1995). Subcellular Localization of and Photosensitization by Protoporphyrin Ix in Human Keratinocytes and Fibroblasts Cultivated with 5-Aminolevulinic Acid. Photochem. Photobiol..

[29] Maitra D., Bragazzi Cunha J., Elenbaas J.S., Bonkovsky H.L., Shavit J.A., Omary M.B. (2019). Porphyrin-Induced Protein Oxidation and Aggregation as a Mechanism of Porphyria-Associated Cell Injury. Cell. Mol. Gastroenterol. Hepatol..

[30] Ozanne H., Toumi H., Roubinet B., Landemarre L., Lespessailles E., Daniellou R., Cesaro A., Ozanne H., Toumi H., Roubinet B. (2020). Laminarin Effects, a β-(1,3)-Glucan, on Skin Cell Inflammation and Oxidation. Cosmetics.

[31] André P., Villain F. (2017). Free Radical Scavenging Properties of Mannitol and Its Role as a Constituent of Hyaluronic Acid Fillers: A Literature Review. Int. J. Cosmet. Sci..

[32] Rajauria G., Ravindran R., Garcia-Vaquero M., Rai D.K., Sweeney T., O’Doherty J. (2021). Molecular Characteristics and Antioxidant Activity of Laminarin Extracted from the Seaweed Species *Laminaria hyperborea,* Using Hydrothermal-Assisted Extraction and a Multi-Step Purification Procedure. Food Hydrocoll..

[33] Chen J., Liu Y., Zhao Z., Qiu J. (2021). Oxidative Stress in the Skin: Impact and Related Protection. Int. J. Cosmet. Sci..

[34] Shin J.-W., Kwon S.-H., Choi J.-Y., Na J.-I., Huh C.-H., Choi H.-R., Park K.-C., Shin J.-W., Kwon S.-H., Choi J.-Y. (2019). Molecular Mechanisms of Dermal Aging and Antiaging Approaches. Int. J. Mol. Sci..

[35] Scholz C.F.P., Jensen A., Lomholt H.B., Brüggemann H., Kilian M. (2014). A Novel High-Resolution Single Locus Sequence Typing Scheme for Mixed Populations of *Propionibacterium acnes* In Vivo. PLoS ONE.

[36] Fitz-Gibbon S., Tomida S., Chiu B.-H., Nguyen L., Du C., Liu M., Elashoff D., Erfe M.C., Loncaric A., Kim J. (2013). *Propionibacterium acnes* Strain Populations in the Human Skin Microbiome Associated with Acne. J. Investig. Dermatol..

[37] Hamblin M.R., Viveiros J., Yang C., Ahmadi A., Ganz R.A., Tolkoff M.J. (2005). *Helicobacter pylori* Accumulates Photoactive Porphyrins and Is Killed by Visible Light. Antimicrob. Agents Chemother..

[38] Mancini S., Imlay J.A. (2015). Bacterial Porphyrin Extraction and Quantification by LC/MS/MS Analysis. Bio Protoc..

[39] van Meerloo J., Kaspers G.J.L., Cloos J., Cree I.A. (2011). Cell Sensitivity Assays: The MTT Assay. Cancer Cell Culture.

[40] Chardon A., Cretois I., Hourseau C. (1991). Skin Colour Typology and Suntanning Pathways. Intern. J. Cosmet. Sci..

[41] Schneider C.A., Rasband W.S., Eliceiri K.W. (2012). NIH Image to ImageJ: 25 Years of Image Analysis. Nat. Methods.

