# Supplementary material for: Seaweed-Derived Extract Targets Porphyr’ageing to Modulate the Visible Signs of Aging in Human Skin
Source: Mar Drugs. 2026 Jun 18;24(6):220. doi: 10.3390/md24060220 (PMC13301944; doi:10.3390/md24060220)
Supplement: Supplementary file 1 [file marinedrugs-24-00220-s001.zip › marinedrugs-4288631-supplementary.pdf]

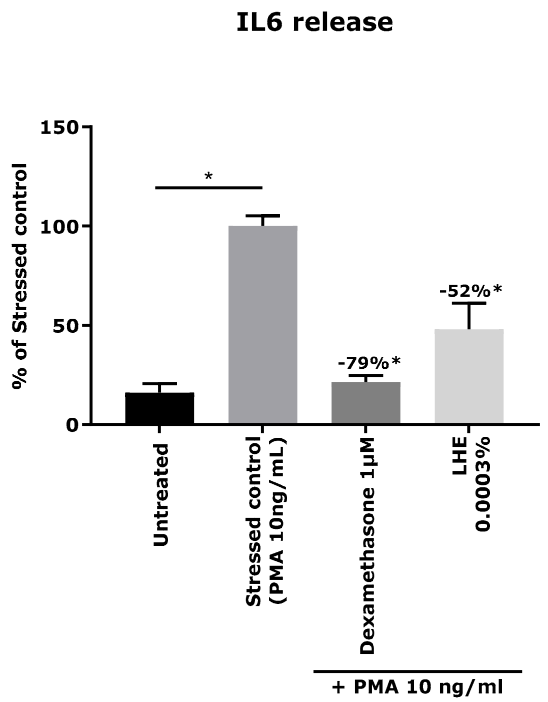

Figure S1. Quantification of IL-6 release in Normal Human Epidermal Keratinocytes (NHEKs) after 24 hours of pro-inflammatory stress mediated by Phorbol 12-myristate 13-acetat at 10ng/mL or in combination with LHE 0.0003%. Results are expressed in % relative to the stressed control mean ± SEM. Statistical analysis was performed using Mann Whitney test. *p<0.05.

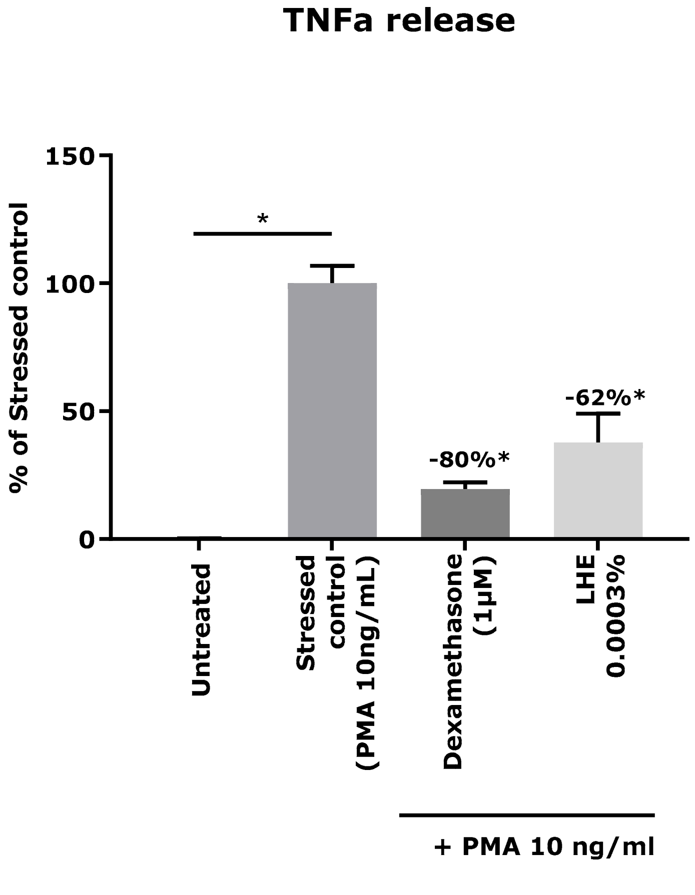

Figure S2. Quantification of TNF-α release in Normal Human Epidermal Keratinocytes (NHEKs) after 24 hours of pro-inflammatory stress mediated by Phorbol 12-myristate 13-acetat at 10ng/mL or in combination with LHE 0.0003%. Results are expressed in % relative to the stressed control mean ± SEM. Statistical analysis was performed using Mann Whitney test. *p<0.05.

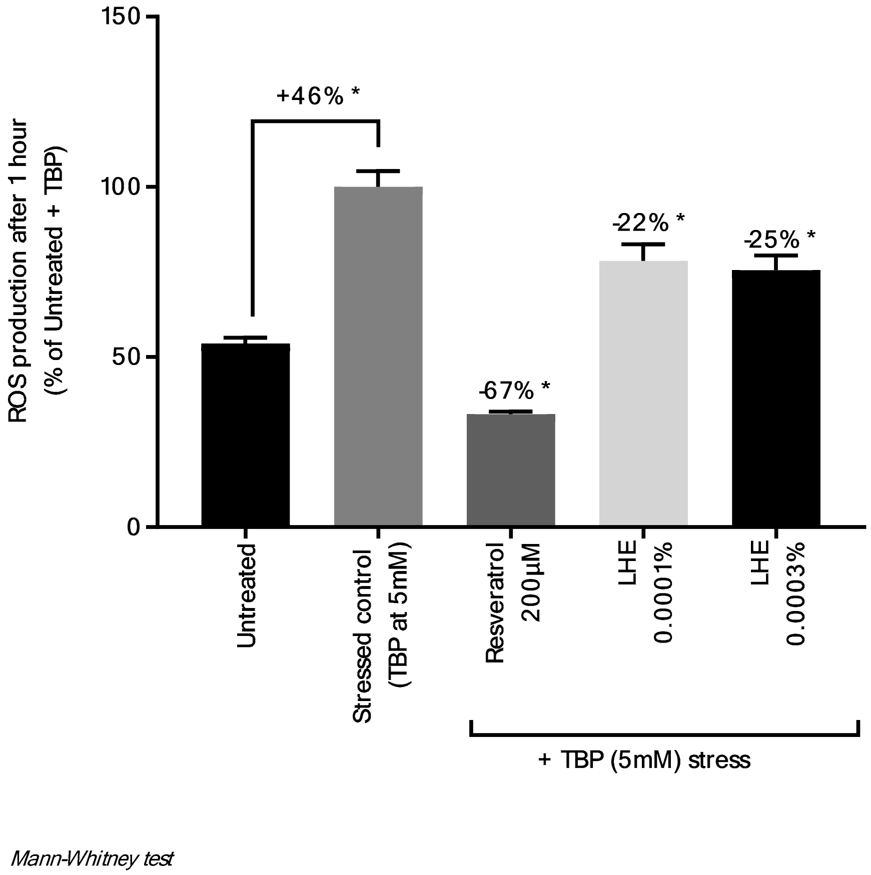

Figure S3. Quantification of intracellular Reactive Oxygen Species (ROS) in normal human skin cells untreated or after oxidative stress mediated by tert-Butyl hydroperoxide solution (TBP) at 5mM or in combination with LHE 0.0003% or 0.0001%. Resveratrol at 200µM was used as positive control. The ROS production was evaluated by measuring the fluorescence generated via the presence of the 2′,7′-Dichlorofluorescin diacetate (DCFH-DA) probe. Results are expressed in % relative to the stressed control mean ± SEM. Statistical analysis was performed using Mann Whitney test. *p<0.05.
